# *ZmEXPB7*, a β-expansin gene, contributes to drought tolerance in *Arabidopsis*

**DOI:** 10.3389/fgene.2025.1688658

**Published:** 2025-09-26

**Authors:** Jing Xiong, Xianqiu Wang, Xueling Ye, Changying Liu, Jialiang Han

**Affiliations:** ^1^ Institute for Advanced Study, Chengdu University, Chengdu, Sichuan, China; ^2^ Key Laboratory of Coarse Cereal Processing, Ministry of Agriculture and Rural Affairs, School of Food and Biological Engineering, Chengdu University, Chengdu, Sichuan, China; ^3^ School of Modern Agriculture, Meishan Vocational and Technical College, Meishan, Sichuan, China; ^4^ Office of Academic Affairs, Chengdu University, Chengdu, Sichuan, China

**Keywords:** ZmEXPB7, drought tolerance, stomatal regulation, reactive oxygen species (ROS), maize

## Abstract

Drought stress poses a significant threat to maize productivity, highlighting the need to elucidate molecular mechanisms underlying drought tolerance. Previous studies identified *ZmLBD33* as a regulator of drought stress responses that interacts with the cell wall-loosening gene *ZmEXPB7*. To elucidate the function of *ZmEXPB7* in drought tolerance, we conducted heterologous expression studies in Arabidopsis. The results demonstrated that *ZmEXPB7* expression was rapidly induced under PEG_6000_-simulated stress, reaching peak levels within 1 h. Overexpression of *ZmEXPB7* in Arabidopsis significantly enhanced drought tolerance, improved root growth, and increased survival rates under osmotic and soil drought conditions. Transgenic plants exhibited reduced water loss, decreased stomatal density, and enhanced stomatal closure. DAB and NBT staining demonstrated that the ROS accumulated in *ZmEXPB7*-overexpressed Arabidopsis. A physiological index assay also revealed that SOD and POD activities in *ZmEXPB7*-overexpressed Arabidopsis were lower than those in wild-type Arabidopsis. These findings indicate that *ZmEXPB7* positively regulates drought tolerance by modulating stomatal aperture and H_2_O_2_ signaling. This study highlights the crucial role of expansin genes in stress adaptation and positions *ZmEXPB7* as a potential target for engineering drought-resilient crops.

## 1 Introduction

Global maize-growing regions are facing increasing threats from drought stress, which has severely impacted maize yields. Abnormal droughts have caused significant yield reductions in America, particularly in areas with light-textured soils (Lobell et al., 2013). In Thailand, climate change has led to prolonged droughts, significantly reducing the suitability of the region for maize cultivation (Amnuaylojaroen et al., 2021). Drought impacts in China’s major maize-producing areas exhibit distinct regional and temporal characteristics. In Northeast China, the limited precipitation during the growing season, with a pronounced concentration in the latter phenological stages, has significantly impaired maize growth and development (Wan et al., 2022). In the Huang-Huai-Hai region, drought conditions have worsened, with precipitation from May 2024 onward being 70% below the annual average. In core production areas such as central Hebei, northern Henan, and southwestern Shandong, approximately 63% of farmland had relative soil water content below 55% in the 0–40 cm soil layer (classified as moderate to severe drought), severely impeding sowing and seedling emergence (Yanping, 2025). The FAO notes that the frequency of such drought events has increased by 34% over the past decade, directly threatening national food security (FAO, 2023).

Drought stress significantly and multifacetedly affects maize growth, development, and yield. First, drought stress directly inhibits maize morphogenesis. Drought leads to reduced plant height, decreased leaf area, and diminished dry matter accumulation. It also delays the development of tassels and ears, prolongs the anthesis-silking interval (ASI), impairs pollination and fertilization, reduces floret differentiation, and increases kernel abortion, ultimately lowering kernel number per ear (Yang et al., 2018; Guo et al., 2021; Wang et al., 2019). Second, drought disrupts maize water balance and photosynthesis, triggering stomatal closure to reduce transpiration while simultaneously lowering photosynthetic efficiency (Tuzet et al., 2010; Mura and Vangimalla, 2020). The accumulation of reactive oxygen species (ROS) exacerbates membrane lipid peroxidation, increases malondialdehyde (MDA) content, and causes abnormal activity of antioxidant enzymes such as superoxide dismutase (SOD) and peroxidase (POD), further damaging cell structures (Faize et al., 2011). During drought stress responses, abscisic acid (ABA) and hydrogen peroxide (H_2_O_2_) serve as critical signaling molecules, with their levels regulated by transcription factors (Zhu, 2016; Kubila et al., 2017; Miller et al., 2010). Drought-induced changes in ABA activate receptors on guard cell membranes, catalyzing H_2_O_2_ production via nicotinamide adenine dinucleotide phosphate (NADPH) oxidase, which in turn activates calcium channels and triggers protein kinase cascades, further regulating anion channels to induce cell water loss and stomatal closure (Kwak et al., 2003; Pei et al., 2000; Zhang et al., 2001).

Expansin protein family represents a crucial group of cell wall-modifying agents that facilitate acid growth through targeted cleavage of hydrogen bonds linking matrix polysaccharides. These proteins are widely present in plants and feature highly conserved domains, including an N-terminal signal peptide, a catalytic domain (DPBB_1, Domain A), and a C-terminal pollen allergen domain (Domain B), with Domain B potentially involved in substrate recognition (Cosgrove, 2000a; Yennawar et al., 2006). Based on sequence homology and phylogenetic relationships, the expansin family is divided into four subfamilies: EXPA (α-expansin), EXPB (β-expansin), EXLA, and EXLB, with EXPA and EXPB being widely present and functionally well-characterized in plants (Kende et al., 2004). Genomic studies reveal significant interspecies variation in the number of Expansin genes: 93 Expansin genes have been identified in maize, including 40 EXPA, 47 EXPB, and 6 EXLA genes, with no EXLB genes detected. These genes are predominantly clustered on chromosomes, with their expansion primarily driven by segmental and tandem duplication events (Jiang et al., 2018). The promoters of Expansin genes are enriched with hormone-responsive elements (e.g., ABA-responsive ABRE and auxin-responsive TGA-element) and stress-responsive elements (e.g., MYB-binding sites), suggesting their involvement in transcriptional regulatory networks governing development and stress responses (Sampedro and Cosgrove, 2005).

Expansin genes exhibit diverse functions in plants, primarily participating in vegetative organs development, pollen tube growth, seed germination, and fruit development, while also playing important roles in responses to biotic and abiotic stresses. Expansin genes promote cell elongation and division by facilitating cell wall loosening: *AtEXPA1* influences asymmetric division of pericycle cells and radial expansion of lateral root primordia, thereby affecting lateral root development in Arabidopsis (Ramakrishna et al., 2019). In Arabidopsis, AtLBD18/AtASL20 directly binds to the *EXPANSINA14 (AtEXPA14)* promoter to activate its expression and promote lateral root growth (Lee and Kim, 2013). In rice, *OsEXPA10* expression in root tips is essential for root cell elongation, and its absence impedes root growth (Che et al., 2016). Heterologous expression of *ClEXPA1* and *ClEXPA2* in tobacco increases pith parenchyma cell size and stem thickness, indirectly affecting cellulose metabolism (Wang et al., 2011). *OsEXPA7* influences yield and quality by participating in the jasmonic acid metabolic pathway (Zhang et al., 2024). *TaEXPA6* affects grain weight and increases yield (Vicentin et al., 2023). *SlEXP1* synergizes with endoglucanase *SlCEL2* to promote fruit softening by enhancing cell wall disassembly (Su et al., 2024). In maize, *ZmEXPB15* regulates kernel size and weight by coordinating nucellus degradation and early endosperm development, with its expression controlled by transcription factors *ZmNAC11* and *ZmNAC29* (Sun et al., 2022).

Additionally, Expansin plays significant role in biotic and abiotic stress responses. *AtEXPA3*, *AtEXPA6*, *AtEXPA8*, *AtEXPA10*, and *AtEXPA16* participate in nematode-induced syncytium formation in roots (Wieczorek et al., 2006). *OsEXPA10* has dual functions: it regulates growth while also participating in biotic stress responses. Overexpression of *OsEXPA10* promotes rice growth but increases susceptibility to brown planthopper infestation and rice blast, whereas knockdown reduces plant height and grain size while enhancing resistance to pests (Tan et al., 2018). In soybean, *GmEXPA11*, which is transcriptionally regulated by *GmPTF1*, interacts with *GmNOD20* to synergistically promote nodule enlargement and enhance nitrogen fixation efficiency (Xing et al., 2025). *GmEXPB2* increases root cortex cell proliferation and expansion, stimulates root hair formation and nodule development, and optimizes root system architecture, thereby enhancing phosphorus acquisition efficiency in soybean (Yang et al., 2021). *TaEXPB23* enhances salt tolerance by improving water retention and lowering osmotic potential in tobacco (Han et al., 2012). *TaEXPA2* interacts with *TaMPS* to stimulate lateral root formation, increase cellular water retention, and mitigate oxidative stress by reducing ROS levels, ultimately conferring drought tolerance in wheat (Yang et al., 2020). In maize, overexpression of *ZmEXPA4* and *ZmEXPA5* alleviates drought-induced increases in the anthesis-silking interval and improves yield (Liu et al., 2021; Tao et al., 2023).

Our previous studies identified ZmLBD33 as a regulator of drought stress responses that interacts with the cell wall-loosening protein ZmEXPB7. To elucidate the function of *ZmEXPB7* in drought tolerance, we conducted heterologous expression studies in Arabidopsis. Our results demonstrated that *ZmEXPB7* expression was rapidly induced under PEG_6000_ stress, reaching peak levels within 1 h. Overexpressing *ZmEXPB7* seedlings displayed significantly drought tolerance in Arabidopsis, which correlated with enhanced root system architecture - including increased root length, surface area, and tip number - facilitating more efficient soil water exploration. The transgenic seedlings also exhibited reduced stomatal density and aperture, resulting in decreased water loss rates. These findings position *ZmEXPB7* as a dual-function regulator that modulates both root morphology and stomatal behavior to enhance drought tolerance. Our study provides mechanistic insights into expansin-mediated stress adaptation and identifies *ZmEXPB7* as a promising target for developing drought-resistant maize cultivars.

## 2 Materials and methods

### 2.1 Expression levels of *ZmEXP7* under drought stress

To investigate the role of *ZmEXP7* under drought stress, maize seedlings at the one-leaf stage were transplanted into Hoagland nutrient solution and cultured until the three-leaf stage. The seedlings were then subjected to drought stress by treating with 20% (w/v) PEG_6000_ dissolved directly in the culture solution (Li et al., 2017; Ma et al., 2018). Roots were collected at 0 h, 1 h, 3 h, 6 h, 12 h, 24 h, and 48 h after PEG_6000_ treatment, immediately frozen in liquid nitrogen, and stored at −80 °C. All samples were collected at the terminal time point. Uniformly grown, healthy maize seedlings were selected for sampling. Each experiment included at least three biological replicates.

### 2.2 RNA extraction and quantitative real-time PCR

Total RNA was extracted following the instructions of the Plant Total RNA Isolation Kit (FOREGENE, RE-05014). Specific primers for *ZmEXPB7* were designed to span intronic regions, based on the B73 reference genome. Quantitative real-time PCR (RT-PCR) was performed using *Zme1F1α* and *Zm18S* as internal reference genes. The reaction system was prepared according to the SYBR Green Fast qPCR Mix kit (ABclonal, RM21203) manual, and RT-PCR amplification was conducted on a Bio-Rad CFX96 PCR instrument following the protocol. The experimental results were analyzed using the 2^−ΔΔCT^ method. All experiments included three biological replicates.

### 2.3 Drought experiments with *ZmEXPB7*-Overexpressing Arabidopsis

The *ZmEXPB7* coding sequence was cloned into the pBI121-mcherry expression vector to generate the *ZmEXPB7*-mcherry vector. The *ZmEXPB7* gene was overexpressed in wild-type Arabidopsis using the floral dip method. After obtaining homozygous seeds, seedlings with high expression levels were selected for phenotypic characterization experiments.

Sterilized seeds of the overexpressing Arabidopsis homozygous lines and wild-type were sown on 0, 200, 250, 300 mM mannitol medium. After vernalization at 4 °C for 72 h, seeds were transferred to short-day conditions for growth.

#### 2.3.1 Germination rate

Radicle emergence was recorded as germination, and the number of germinated seeds for each genotype was counted until no further changes were observed per 12 h under each stress condition.

#### 2.3.2 Survival rate

After germination 5 days, seedlings were scored for greening under different stress treatments. Albino seedlings and ungerminated seeds were classified as dead, while green seedlings were counted as survival.

#### 2.3.3 Mannitol stress assay

After 5 days of growth on normal medium, well-grown and uniform lines were transplanted to freshly prepared 0, 200 mM, 250 mM, 300 mM mannitol medium for vertical culture. After 7 days, differences between genotypes were observed, photographed, and root images were acquired using an EPSON 11000XL root scanner. Root traits were analyzed using WinRhizo Pro2013 software.

#### 2.3.4 Drought stress assay

Seven-day-old seedlings grown on normal medium were transplanted into soil. After 1 month of growth in soil, the growth of different genotypes under normal conditions was photographed and recorded. Seedlings from different genotypes were subjected to a 7-day water-withholding period, after which clear wilting symptoms were recorded. Survival rates were quantified after 3 days of rewatering.

### 2.4 Water loss rate measurement in Arabidopsis

The fresh weight of the aerial parts was measured using one-month-old Arabidopsis seedlings. The seedlings were then placed on a laboratory bench, and their weights were recorded at predetermined time points (0.5 h, 1 h, 2 h, 3 h). The rate of water loss was determined at designated time intervals using the following equation: water loss rate (%) = [(W_0_ - W_t_)/W_0_] × 100.

### 2.5 Stomatal aperture measurement in Arabidopsis

The middle portion of the fourth leaf from one-month-old Arabidopsis plants was placed on a laboratory bench and dehydrated for 1 h. Both normal and dehydrated Arabidopsis leaves were fixed in Carnoy’s fixative (absolute ethanol: glacial acetic acid = 3:1) for 24 h. The leaves were then dehydrated sequentially in 30%, 50%, 70%, 80%, 85%, 90%, 95%, and 100% ethanol for 30 min each. The dehydrated leaves were placed in clearing solution (chloral hydrate: water: glycerol = 8:3:1) until transparent. Stomata were observed under a light microscope, and their length and width were measured using ImageJ. Stomatal aperture was expressed as the ratio of stomatal width to length.

### 2.6 NBT and DAB staining

Arabidopsis leaves were immersed in NBT staining solution (0.01 g NBT powder dissolved in 10 mL of 50 mM phosphate buffer, pH 7.8) or DAB staining solution (0.1% DAB in HCl solution, pH 3.8, protected from light). After vacuum infiltration for 30 min, the leaves were kept in the dark at 22 °C for 10 h. The leaves were then decolorized in decolorizing solution (acetic acid: glycerol: ethanol = 1:1:3) by boiling for 5 min and stored in 95% ethanol for slide preparation and observation.

### 2.7 H_2_O_2_ content measurement

Fresh tissue (0.1 g) was flash-frozen in liquid nitrogen and ground to a powder. After adding 1 mL of 0.1% TCA and mixing thoroughly, the sample was centrifuged at 4 °C and 12,000 r/min for 15 min. Then, 500 µL of the supernatant was mixed with an equal volume of PBS buffer and 1 mL of 1 M KI solution. The mixture was incubated at 30 °C in the dark with shaking (150 r/min) for 1 h, and the absorbance was measured at 390 nm. A standard curve was prepared using 300 μmol/L H_2_O_2_ as the stock solution. H_2_O_2_ content (µmol/g FW) was calculated using: C × V_t_/(FW × V_1_). Measurements were performed with three biological replicates, each containing three technical replicates.

### 2.8 Antioxidant enzyme activity assay

#### 2.8.1 Enzyme extraction

Fresh leaf samples (0.1 g) were homogenized in liquid nitrogen and extracted with 1 mL of ice-cold 50 mM phosphate buffer (pH 7.8) containing 1% PVP, 2 mM DTT, and 0.1 mM EDTA. After incubation on ice for 10 min, the homogenate was centrifuged at 12,000 × g for 15 min at 4 °C, and the supernatant was used for enzyme assays.

#### 2.8.2 Superoxide dismutase (SOD) activity

The reaction mixture (3 mL) contained 50 mM phosphate buffer (pH 7.8), 130 mM methionine, 750 μM NBT, 100 μM EDTA-Na_2_, 20 μM riboflavin, and 50 μL enzyme extract. One control tube was wrapped in aluminum foil and kept in the dark throughout the experiment, while another and the sample tubes were illuminated at an intensity of 4,000 lx for 20 min using fluorescent lamps. Absorbance was measured at 560 nm. One unit (U) of SOD activity was defined as the amount of enzyme required to inhibit 50% of the photochemical reduction of NBT.

#### 2.8.3 Peroxidase (POD) activity

The reaction mixture (3 mL) contained 0.3% (v/v) H_2_O_2_, 0.2% (v/v) guaiacol, 50 mM phosphate-buffered saline (PBS, pH 7.0), and 50 μL of enzyme extract. The reaction was initiated by adding the enzyme extract, and the increase in absorbance at 470 nm was recorded every 30 s for 5 min at 25 °C. One unit (U) of POD activity was defined as an increase of 0.01 in absorbance per minute under the assay conditions.

#### 2.8.4 Catalase (CAT) activity

The reaction mixture (3 mL) contained 0.3% (v/v) H_2_O_2_ in 50 mM phosphate buffer (pH 7.0) and 50 μL of enzyme extract. The decrease in absorbance at 240 nm was monitored at 25 °C for 3 min, with readings taken at 30-s intervals. One unit (U) of CAT activity was defined as a decrease of 0.1 in absorbance per minute.

Enzyme activities were expressed as U/(g min) (POD, CAT) or U/g (SOD) based on fresh weight. All assays were performed with three biological and technical replicates.

### 2.9 Yeast two-hybrid assay

The *ZmEXPB7* coding sequence was cloned into the pGBKT7 vector (EXPB7-BD) and the full-length fragment of *ZmLBD33* was cloned into the pGADT7 vector (LBD33-AD). Yeast cells harboring the *ZmEXPB7*-BD and *ZmLBD33*-AD, or *ZmEXPB7*-BD and pGADT7, or *ZmLBD33*-AD and pGBKT7 were respectively diluted to three concentrations and grown on nonselective (SD/-Trp/-Leu) or selective (SD/-Trp/-Leu/-His/-Ade) medium. A pGBKT7-53 and pGADT7-T combination was used as a positive control. A pGBKT7-Lam and pGADT7-T combination was used as a negative control.

### 2.10 Bimolecular fluorescence complementation (BiFC) assay

The *ZmEXPB7* coding sequence was cloned into the pXYc104 vector (yielding *ZmEXPB7*-104, containing the C-terminal fragment of the fluorescent protein), while the *ZmLBD33* gene was inserted into the pXYn106 vector (producing *ZmLBD33*-106, harboring the N-terminal fragment). For transient co-expression, both constructs were introduced into Agrobacterium tumefaciens strain GV3101 and co-infiltrated into Nicotiana benthamiana leaves. Following 36–48 h of incubation under controlled conditions, reconstituted fluorescence was visualized using confocal laser scanning microscopy.

### 2.11 Co-immunoprecipitation (Co-IP)

Agrobacterium tumefaciens strains harboring either *ZmEXPB7*-mCherry or *ZmLBD33*-eGFP/pCAMBIA2300 (control) plasmids were mixed (1:1) and co-infiltrated into Nicotiana benthamiana leaves. After 36 h, infiltrated leaf tissues were flash-frozen, homogenized in ice-cold extraction buffer (50 mM Tris-HCl pH 7.9, 120 mM NaCl, 5 mM EDTA, 1 mM PMSF, 10 mM DTT, 0.1% NP-40, protease inhibitors), and centrifuged (12,000 × g, 15 min, 4 °C). The supernatant was incubated with anti-GFP magnetic beads (Abclonal, AE079) for 2 h at 4 °C. Beads were washed, resuspended in 2× SDS loading buffer, and boiled (95 °C, 15 min). Input and immunoprecipitated samples were analyzed by immunoblotting using anti-GFP (Abcam, ab290) and anti-mCherry (ImmunoWay, YM3212) antibodies.

### 2.12 Western blot analysis

Proteins were separated by SDS-PAGE using a 10% separating gel, followed by electrophoretic transfer onto a methanol-activated PVDF membrane using a semi-dry transfer system. The membrane was blocked with 5% skim milk for 2 h at room temperature, then incubated overnight at 4 °C with primary antibody (diluted as recommended). After TBST washes, HRP-conjugated secondary antibody (1:10,000) was applied for 2 h at room temperature. Protein bands were visualized using ECL substrate and chemiluminescence detection after rigorous TBST washing steps.

## 3 Results

### 3.1 Interaction between ZmLBD33 and ZmEXPB7 proteins

**FIGURE 1:**
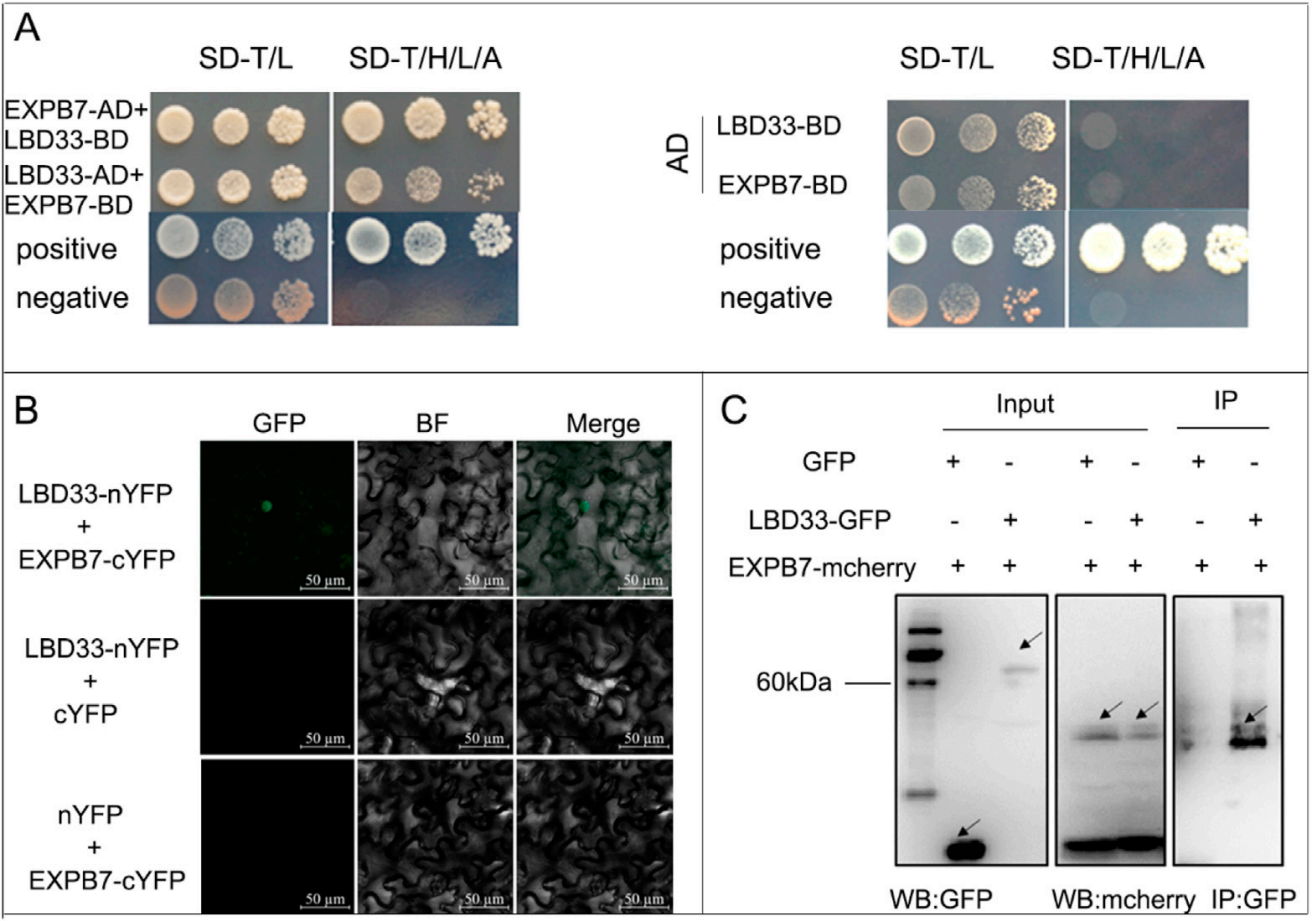
Interaction between the ZmLBD33 and ZmEXPB7. **(A)** ZmLBD33 and ZmEXPB7 interactions in the yeast two-hybrid assay. Yeast cells co-transformed with ZmLBD33-AD and ZmEXPB7-BD were grown on SD/–Trp/–His/–Leu/Ade medium for 5 days at 30 °C. **(B)** Bimolecular fluorescence complementation (BiFC) assay confirming the interaction between ZmEXPB33 and ZmEXPB7 in *Nicotiana benthamiana* leaves. YFP fluorescence was observed 48 h after agroinfiltration. Scale bar = 50 µm. **(C)** Co-immunoprecipitation (Co-IP) assay detecting the interaction between ZmLBD33 and ZmEXPB7 *in vivo*. Proteins were extracted from leaves expressing ZmLBD33-GFP and ZmEXPB7-mcherry and immunoprecipitated with anti-GFP beads, followed by immunoblotting using anti-mcherry antibody.

Yeast library screening revealed that ZmEXPB7 is an interacting protein of ZmLBD33. To validate whether ZmEXPB7 indeed interacts with ZmLBD33, *ZmEXPB7*-BD was co-transformed with *ZmLBD33*-AD or the pGADT7 empty vector into yeast Y2HGold strain. On double dropout (-Trp/-Leu) medium, the growth status of the co-transformants was similar to that of the positive and negative controls. On quadruple dropout (-Trp/-Leu/-His/-Ade) medium, yeast strains co-transformed with *ZmEXPB7*-BD and *ZmLBD33*-AD activated the expression of the downstream reporter genes His and Ade, growing normally like the positive control. This confirmed the interaction between ZmEXPB7 and ZmLBD33 (Figure 1A).

To validate the interaction between ZmLBD33 and ZmEXPB7 in plant, the *ZmEXPB7*-cYFP construct was co-infiltrated with Agrobacterium carrying *ZmLBD33*-nYFP into tobacco leaves at a 1:1 ratio. After 36 h, green fluorescence signals were observed under a microscope. Fluorescence signals were detected in the nuclei of tobacco leaf cells co-expressing ZmLBD33-nYFP and ZmEXPB7-cYFP (Figure 1B). In contrast, no fluorescence was observed when ZmLBD33-nYFP was co-infiltrated with cYFP or nYFP was co-infiltrated with ZmEXPB7-cYFP. The BiFC assay demonstrated that ZmLBD33 interacts with ZmEXPB7 proteins in planta, and the interaction occurs in the nucleus.

To further confirm the interaction between ZmLBD33 and ZmEXPB7 in living plant cells, the *ZmEXPB7*-mcherry construct was transformed into Agrobacterium and co-infiltrated with *ZmLBD33*-eGFP Agrobacterium at a 1:1 ratio. A negative control was prepared by co-infiltrating pCAMBIA2300-eGFP with *ZmEXPB7*-mcherry. After 36 h of infiltration, fluorescence was observed, and samples were collected for protein extraction under non-denaturing conditions. Co-immunoprecipitation was performed using GFP antibody, followed by Western blot detection with mcherry and GFP antibodies. As shown in the figure (Figure 1C), mcherry antibody detected bands corresponding to ZmEXPB7-mcherry, and GFP antibody detected bands corresponding to GFP and ZmLBD33-GFP, confirming successful expression of both proteins. In the samples immunoprecipitated with GFP antibody-conjugated beads, mcherry antibody detected ZmEXPB7-mcherry only in the ZmLBD33-GFP sample, with no bands detected in the pCAMBIA2300-GFP negative control, ruling out nonspecific binding between ZmEXPB7-mcherry and GFP. These results further demonstrate the interaction between ZmLBD33 and ZmEXPB7 in planta.

### 3.2 Expression pattern analysis of *ZmEXPB7*

**FIGURE 2:**
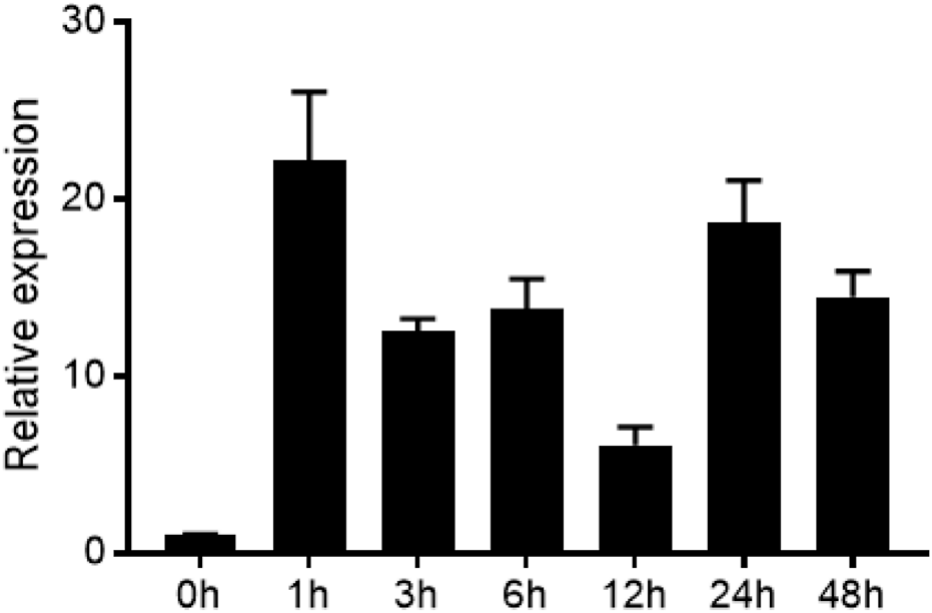
Expression level of *ZmEXPB7* under drought stress.

Given the established protein-protein interaction between ZmEXPB7 and ZmLBD33, we sought to determine whether these proteins exhibit functional conservation in their biological roles. Using qRT-PCR with *Zme1F1α* and *Zm18S* as the internal reference, we analyzed the expression pattern of *ZmEXPB7* under PEG_6000_-simulated drought stress at different time points. *ZmEXPB7* expression was significantly induced under PEG_6000_ stress, peaking at 1 h post-treatment (Figure 2), indicating its responsiveness to osmotic stress.

Expression levels of RNA transcripts were quantified under PEG_6000_ treatment using quantitative real-time PCR, with *Zme1F1α* and *Zm18S* as internal reference genes. Data were analyzed via the 2^−ΔΔCT^ method and are presented as means ± SD (n = 3).

### 3.3 Drought resistance of *ZmEXPB7*-Overexpressing Arabidopsis

**FIGURE 3:**
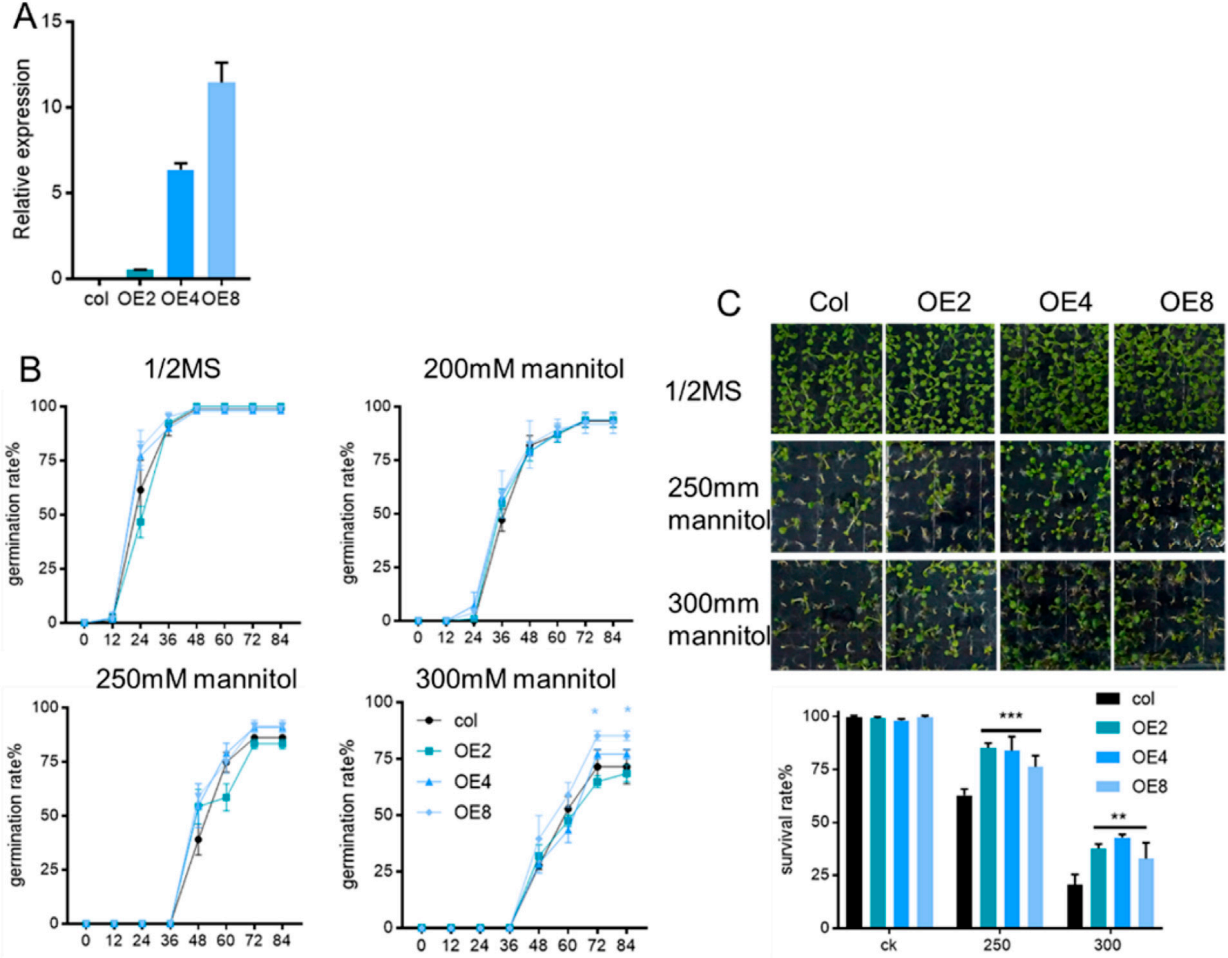
Germination and survival rate of *ZmEXPB7* overexpressing Arabidopsis under different concentration mannitol. **(A)** Expression level of *ZmEXPB7* overexpressed Arabidopsis lines; **(B)** Germination rate of *ZmEXPB7* overexpression Arabidopsis under different concentration mannitol; **(C)** Survival rate of *ZmEXPB7* overexpression Arabidopsis under different concentration mannitol. Each experiment had three repeats, each repeat used 70 seeds. All data represent means ± SD. Asterisks represented the significance compare to wild type by using one-way ANOVA, *, p < 0.05, **, p < 0.01, ***, p < 0.001.

The *ZmEXPB7*-mcherry expression vector was transformed into wild-type Arabidopsis via floral dip method. Kanamycin screening identified 12 positive transformants. RNA was extracted to screen for high-expression lines, and three lines with high expression levels (OE2, OE4, OE8) were chosen for further studies (Figure 3A).

We investigated the resistance of *ZmEXPB7*-overexpressing and wild-type Arabidopsis to mannitol-induced osmotic stress during seed germination and seedling stages. Elevated mannitol concentrations induced a progressive inhibition of *Arabidopsis thaliana* seed germination, resulting in a statistically significant delay in germination (Figure 3B). The germination rates of *ZmEXPB7*-overexpressing and wild-type Arabidopsis showed no significant differences on normal 1/2 MS medium or 1/2MS + 200 mM mannitol medium. However, on 1/2MS + 250 mM and 300 mM mannitol media, *ZmEXPB7*-overexpressing Arabidopsis exhibited higher germination rates than wild-type seedlings, although only OE8 reached statistical significance (Figure 3B).

Five days after germination, the number of normally developed seedlings and albino seedlings was counted on mannitol stress media, with albino seedlings and ungerminated seeds recorded as dead. Under normal conditions, no significant difference in survival rates was observed between *ZmEXPB7*-overexpressing *Arabidopsis thaliana* and wild-type seedlings. However, in half MS supplemented with 250 mM and 300 mM mannitol, the survival rate of *ZmEXPB7*-overexpressing lines showed a statistically significant increase compared to wild-type (Figure 3C). Notably, under 300 mM mannitol stress, the survival count of *ZmEXPB7*-overexpressing seedlings was approximately two-fold higher than that of wild-type seedlings.

**FIGURE 4:**
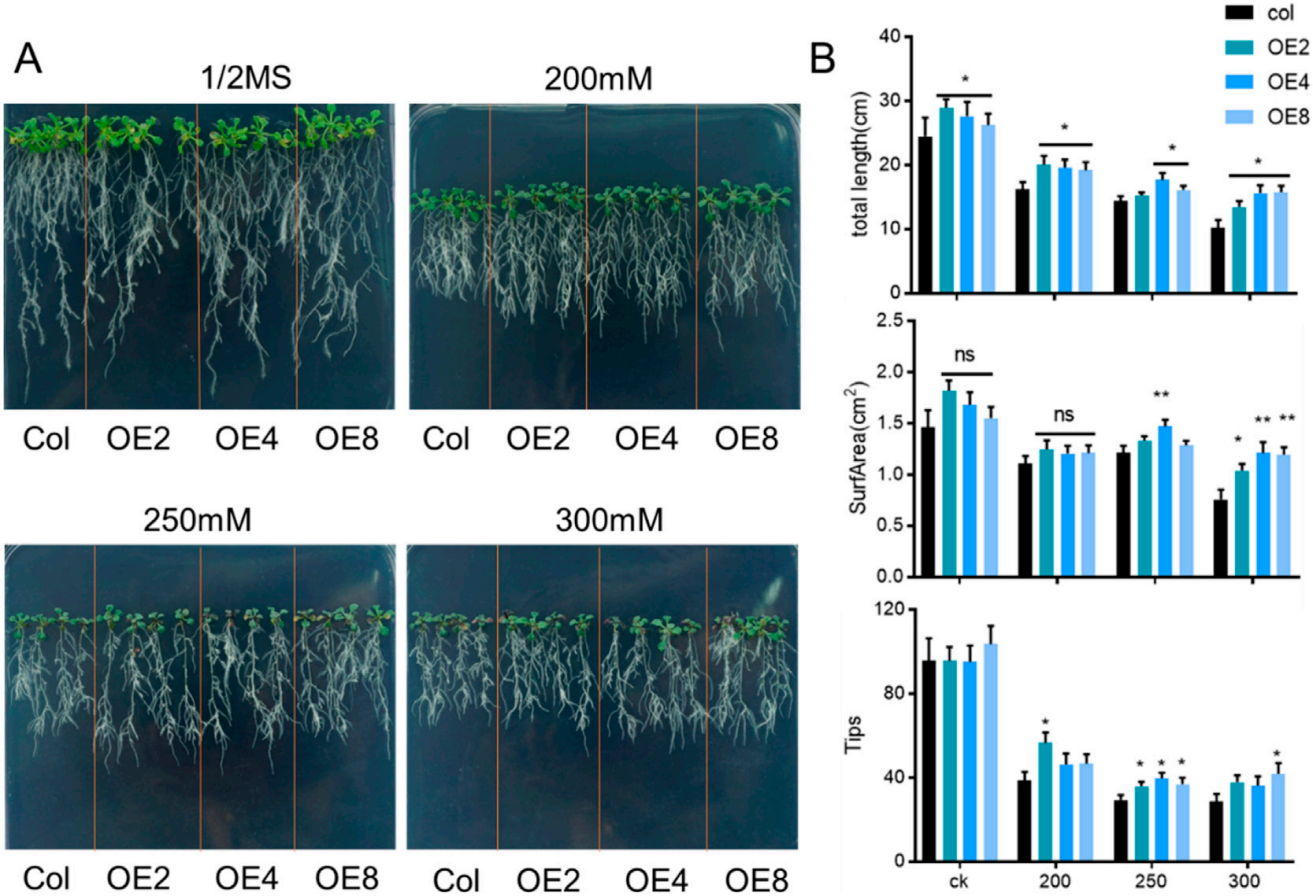
Phenotype of *ZmEXPB7* overexpressed Arabidopsis under different mannitol treatment. **(A)** Phenotype of *ZmEXPB7* overexpressed Arabidopsis under normal condition, 200 mM, 250 mM, 300 mM mannitol concentration; **(B)** Data analysis of total root length, root surf area, root tips under normal condition, 200 mM, 250 mM, 300 mM mannitol in *ZmEXPB7* overexpressed Arabidopsis. Each experiment had three repeats, each time had at least 12 seedlings. All data represent means ± SD. Asterisks represented the significance compare to wild type by using one-way ANOVA, *, p < 0.05, **, p < 0.01, ***, p < 0.001.

To further observe the phenotypic changes of *ZmEXPB7*-overexpressing Arabidopsis under mannitol stress, 5-day-old seedlings grown on normal medium were transferred to mannitol-containing media for 7 days. Analysis of total root length, root surface area, and root tip number revealed that *ZmEXPB7*-overexpressing Arabidopsis exhibited longer roots than wild-type under both normal and mannitol stress conditions. No significant differences in root surface area or tip number were observed on normal or 200 mM mannitol. However, under 250 mM and 300 mM mannitol stress, *ZmEXPB7*-overexpressing Arabidopsis showed significantly higher root surface area and tip numbers than wild-type seedlings, indicating enhanced resistance to high osmotic stress (Figure 4).

**FIGURE 5:**
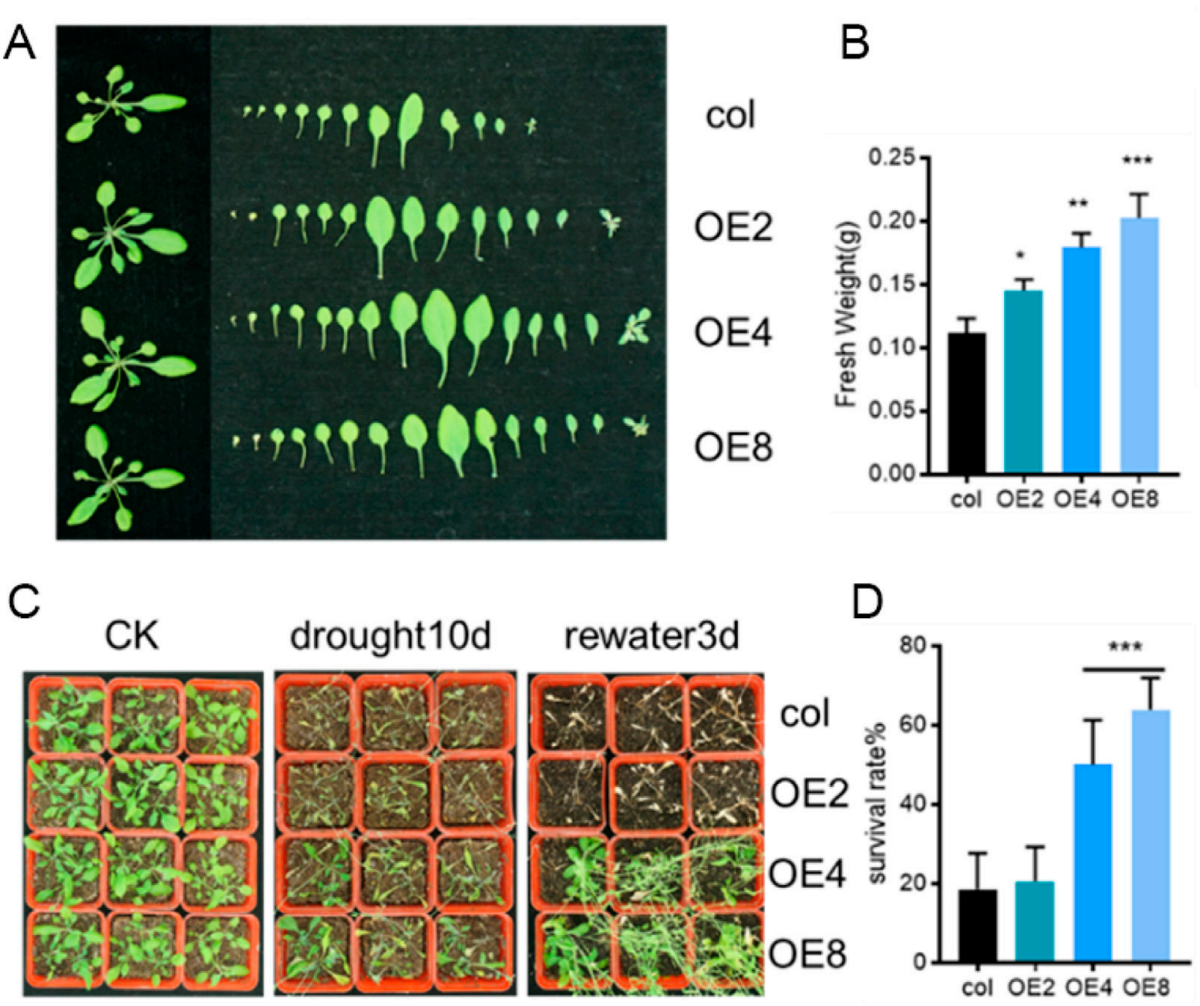
Decrease of stomatal number and aperture contributed to the tolerant of *ZmEXPB7* overexpressed Arabidopsis. **(A,B)** Phenotype and fresh weight of wild-type and *ZmEXPB7*-overexpressing Arabidopsis lines under normal growth conditions. Data represent mean ± SE (n = 12). **(C,D)** Phenotype and survival rate of wild-type and *ZmEXPB7*-overexpressing lines after drought stress treatment. Drought stress was applied by withholding water for 7 days followed by rewatering for 3 days. Survival rates were calculated as the percentage of seedlings recovering after rewatering. Data represent mean ± SE (n = 3). Statistical significance was determined by one-way ANOVA; asterisks indicate significant differences compared with WT (*, p < 0.05, **, p < 0.01, ***, p < 0.001).

To further confirmed the drought resistance of *ZmEXPB7*-overexpressing Arabidopsis, overexpressing and wild-type seedlings were grown in soil for 4 weeks before water was withheld. Phenotypes were observed until wilting occurred, and survival rates were recorded 3 days after rehydration. After 4 weeks of growth, *ZmEXPB7*-overexpressing Arabidopsis exhibited larger rosettes and more leaves than wild-type, with significantly higher aerial fresh weight (Figures 5A,B). At 7 days of drought stress, wild-type leaves showed wilting earlier than *ZmEXPB7*-overexpressing Arabidopsis. After 3 days of rehydration, wild-type survival was only 20%, while *ZmEXPB7*-overexpressing lines (except OE2) showed significantly higher survival rates (Figures 5C,D). Together, these results demonstrate that *ZmEXPB7* overexpression enhances drought tolerance under both osmotic and soil drought stress.

### 3.4 *ZmEXPB7* decreased the water loss rate by reducing the stomatal density and aperture

**FIGURE 6:**
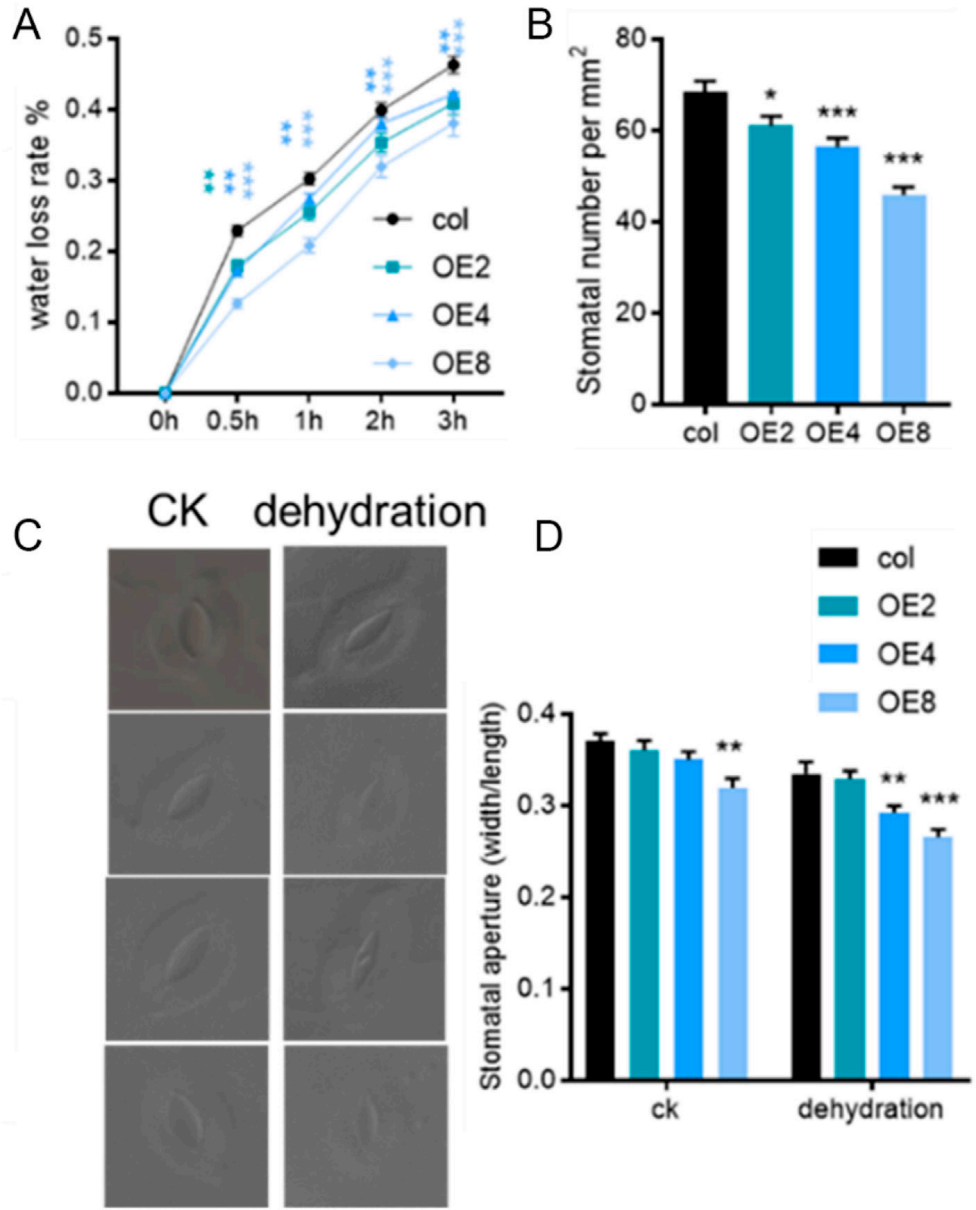
Water loss rate, stomatal number and aperture of leaves in *ZmEXPB7* transgenic Arabidopsis. **(A)** Water loss rate at different time points. **(B)** Stomatal number of fourth leaf medium. **(C,D)** Stomatal aperture of leaves after detaching 1 h. Asterisks represented the significance compare to wild type by using one-way ANOVA, *, p < 0.05, **, p < 0.01, ***, p < 0.001.

To elucidate the relationship between drought resistance and water loss in *ZmEXPB7*-overexpressing Arabidopsis, detached leaves from 4-week-old seedlings were used for water loss rate measurement. Wild-type leaves showed higher water loss rates at all time points than *ZmEXPB7*-overexpressing leaves (Figure 6A). Analysis of stomatal density and aperture in the middle of the fourth leaf revealed that *ZmEXPB7*-overexpressing Arabidopsis had lower stomatal density and significantly smaller stomatal aperture after 1 h of detachment than wild-type (Figures 6B–D), suggesting that reduced stomatal density and aperture slowed water loss, contributing to drought tolerance.

### 3.5 Overexpression of *ZmEXPB7* improved ROS accumulation in Arabidopsis

**FIGURE 7:**
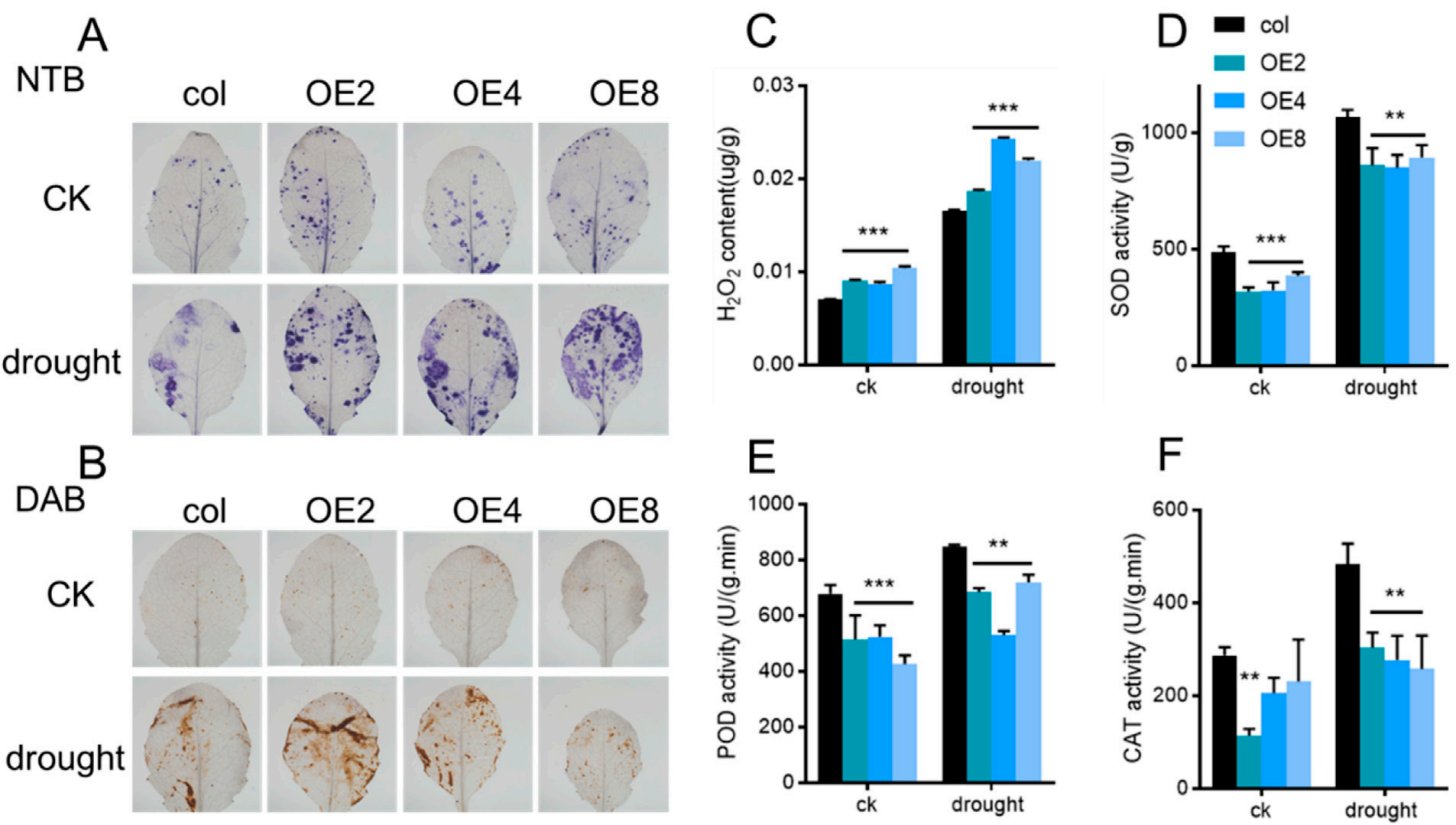
Staining ROS and determination antioxidant enzyme of leaf in *ZmEXPB7* overexpressed Arabidopsis. **(A,B)** NBT and DAB staining of fourth leaf in *ZmEXPB7* overexpressed Arabidopsis; **(C)** H_2_O_2_ content measurement in *ZmEXPB7* overexpressed Arabidopsis leaves. **(D–F)** Enzyme activity of SOD, POD and CAT. Asterisks represented the significance compare to wild type by using one-way ANOVA, *, p < 0.05, **, p < 0.01, ***, p < 0.001.

Drought stress often accompanies reactive oxygen species (ROS) production. NBT and DAB staining were used to detect H_2_O_2_ levels in *ZmEXPB7*-overexpressing and wild-type leaves, with darker staining indicating higher H_2_O_2_ accumulation. NBT and DAB staining was darker in *ZmEXPB7*-overexpressing than wild-type leaves (Figures 7A,B), with more pronounced differences under drought stress. KI-based H_2_O_2_ quantification confirmed that *ZmEXPB7*-overexpressing Arabidopsis accumulated more H_2_O_2_ than wild-type under both normal and drought conditions (Figure 7C). Concurrently, antioxidant enzyme (SOD, POD, CAT) activities were lower in *ZmEXPB7*-overexpressing lines, potentially explaining their higher H_2_O_2_ levels (Figures 7D–F). H_2_O_2_ may act as a signaling molecule to induce stomatal closure.

## 4 Disccusion

Expansin is cell wall-loosening proteins widely involved in cell wall modification and play critical roles in plant growth and development, including vegetative growth (Wang et al., 2011), root elongation (Guo et al., 2011; Lin et al., 2011),flower development (Cosgrove, 2000b),fruit ripening and softening (Shi et al., 2021), and seed yield enhancement (Bae et al., 2014). Heterologous expression of Cunninghamia lanceolata *ClEXPA1* and *ClEXPA2* in tobacco increases plant height, stem thickness, leaf number, carpel thickness, pith parenchyma cell size, and xylem cell wall thickness, directly or indirectly affecting cellulose metabolism (Wang et al., 2011). *OsEXPA10* is essential for root tip elongation (Che et al., 2016),while soybean *GmEXPB2* increases the size and number of cortex cells in root meristem and elongation zones, enhances root hair density, modifies root architecture, and promotes nodule growth and development (Li et al., 2015). In this study, heterologous expression of *ZmEXPB7* in Arabidopsis significantly increased total root length (Figure 4), suggesting that *ZmEXPB7* plays a critical role in promoting root development. The altered root architecture may enhance drought tolerance through two potential mechanisms: (1) well-developed roots can penetrate deeper soil layers, thereby improving water uptake efficiency; and (2) an expanded root surface area facilitates more efficient water exchange at the root-soil interface under drought conditions. These morphological adaptations collectively contribute to enhanced drought tolerance from a root system perspective.

Beyond their established roles in modulating plant growth and development, expansin proteins are increasingly recognized as key players in abiotic stress responses. Previous studies have demonstrated that certain expansins, such as *TaEXPB23* and *TaEXPA2*, contribute to drought tolerance primarily through water retention capacity (Han et al., 2012; Yang et al., 2020). Similarly, overexpression of *ZmEXPA4* and *ZmEXPA5* in maize has been shown to mitigate drought-induced yield loss by shortening the anthesis-silking interval (Liu et al., 2021; Tao et al., 2023). In contrast, our study provides compelling evidence that *ZmEXPB7* confers drought resistance via a novel dual mechanism encompassing both root remodeling and direct stomatal regulation. *ZmEXPB7* expression was rapidly induced by PEG-simulated drought stress, peaking within 1 h of treatment (Figure 2), indicating its involvement in early drought signaling. Transgenic maize overexpressing *ZmEXPB7* exhibited significantly enhanced survival under both osmotic and drought conditions (Figures 3C, 4, 5C), consistent with the general protective role of expansins. However, we further uncovered a previously unreported function of *ZmEXPB7* in stomatal regulation. Overexpression lines showed reduced stomatal density and aperture (Figure 6), resulting in decreased water loss from detached leaves. While previously reported drought-tolerant expansins primarily confer stress resistance through a single mechanism centered on root system modulation, our study uniquely demonstrates that *ZmEXPB7* exerts its drought-tolerance function via a dual regulatory pathway involving both root architecture improvement and stomatal regulation. This distinction marks a key innovation compared to existing research on expansin-mediated drought responses.

We propose that *ZmEXPB7* may influence stomatal formation and kinetics by modulating the mechanical properties of guard cell walls, potentially through interactions with key transcriptional regulators of stomatal development, such as *SPCH (SPEECHLESS)* and *MUTE* (Xiang et al., 2021). These genes are central to the initiation and progression of stomatal lineage cells, and their expression or activity may be indirectly affected by expansin-mediated cell wall modifications. This hypothesized link between expansin action and stomatal developmental programs represents a significant conceptual advance, distinguishing *ZmEXPB7* from other drought-responsive expansins that function primarily through root-based mechanisms. Thus, our work not only identifies *ZmEXPB7* as a critical regulator in maize drought response but also provides new insights into the multifaceted mechanisms by which expansins coordinate stress resilience at both the root and leaf levels. Notably, under drought stress, *ZmEXPB7*-overexpressing plants exhibited unique redox characteristics: elevated H_2_O_2_ levels but reduced SOD, POD, and CAT antioxidant enzyme activities (Figure 7). This seemingly paradoxical phenomenon may reflect the dual role of ROS signaling, where H_2_O_2_ in this context may act more as a secondary messenger to activate stress response pathways, while the ZmEXPB7-ZmLBD33 module may finely regulate ROS homeostasis to balance stress responses and growth inhibition.

The *LBD* gene family is a plant-specific group of transcription factors involved in development and abiotic stress responses. *AtLBD18* plays a crucial role as a transcriptional activator in promoting lateral root formation in *Arabidopsis* by directly regulating *EXPANSIN* genes. Studies demonstrate that AtLBD18 binds to the *EXPANSIN14 (AtEXP14)* promoter, activating its expression to facilitate lateral root emergence (Kim and Lee, 2013). Additionally, AtLBD18, along with its homolog AtASL20, upregulates *EXPANSINA17 (AtEXP17)* during auxin signaling, further enhancing lateral root development (Lee and Kim, 2013). These findings highlight LBD18’s central role in the gene regulatory network of lateral root formation, where it directly modulates *EXPANSIN* family members, including *AtEXP14* and *AtEXP17*, to coordinate cell wall loosening and root primordia emergence (Lee et al., 2013). This study confirmed the interaction between ZmLBD33 and ZmEXPB7 through yeast two-hybrid and bimolecular fluorescence complementation assays (Figure 1), indicating their collaborative involvement in maize drought stress responses. However, the mechanistic details of their joint action in drought responses require further investigation. Moreover, *ZmEXPB7* overexpression produced multiple beneficial phenotypes: promoting root development, optimizing stomatal behavior, and enhancing cell wall mechanical strength. The synergistic effects of these traits ultimately improved overall drought tolerance, demonstrating *ZmEXPB7*’s potential as a preferred target for drought-resistant maize breeding and providing important theoretical foundations for crop drought resistance improvement.

## 5 Conclusion

In conclusion, our study reveals that *ZmEXPB7* enhances drought tolerance in *Arabidopsis* through dual mechanisms: improving root architecture for efficient water uptake and reducing stomatal density to minimize water loss. The interaction between ZmEXPB7 and ZmLBD33, along with elevated H_2_O_2_ levels and altered antioxidant enzyme activities, suggests a role for ROS signaling in stress adaptation. These findings highlight *ZmEXPB7* as a key regulator of drought responses and a promising target for breeding drought-resistant maize varieties. Subsequent efforts are focused on validating these findings in maize through CRISPR-Cas9 gene editing and overexpression studies, which will further elucidate its agronomic potential.

## Data Availability

The original contributions presented in the study are included in the article/Supplementary Material, further inquiries can be directed to the corresponding authors.
