# Supplementary material for: ZmEXPB7, a β-expansin gene, contributes to drought tolerance in Arabidopsis
Source: Front Genet. 2025 Sep 26;16:1688658. doi: 10.3389/fgene.2025.1688658 (PMC12510680; doi:10.3389/fgene.2025.1688658)

Supplement

The interaction between EXPB7 and LBD33 completely scans the entire original gel.

The content of markers is much higher than that of samples, and the exposure is very strong.


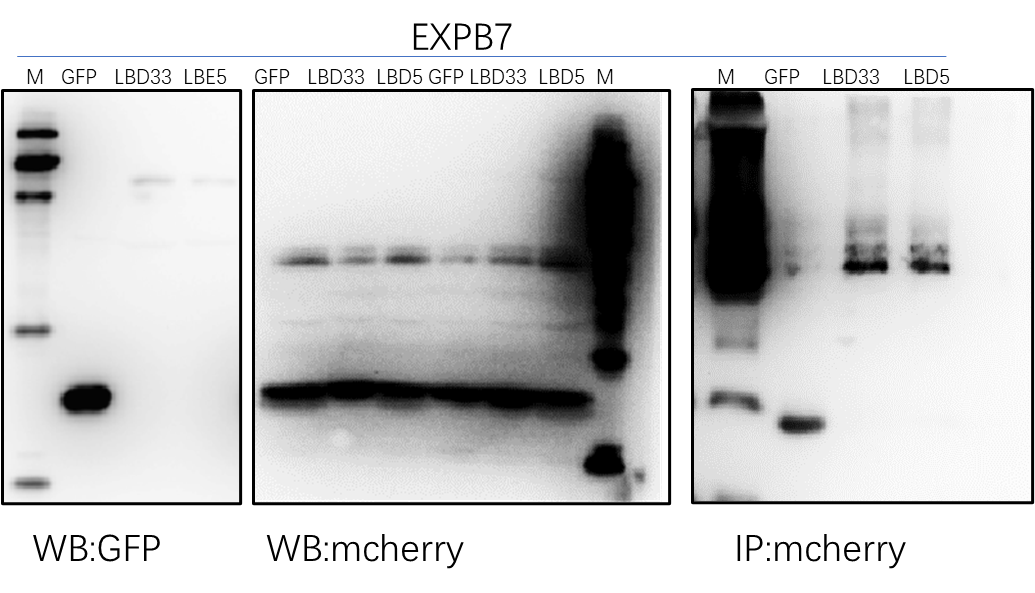

Supplement: Supplementary file 1 [file Table1.docx]
